# Early warning score validation methodologies and performance metrics: a systematic review

**DOI:** 10.1186/s12911-020-01144-8

**Published:** 2020-06-18

**Authors:** Andrew Hao Sen Fang, Wan Tin Lim, Tharmmambal Balakrishnan

**Affiliations:** 1 Bedok Polyclinic, SingHealth Polyclinics, Singapore, Singapore; 2 Department of Internal Medicine, Singapore General Hospital, Singapore, Singapore; 3 Department of Internal Medicine, Singapore General Hospital, Singapore, Singapore

**Keywords:** Early warning score, Validation, Methodology

## Abstract

**Background:**

Early warning scores (EWS) have been developed as clinical prognostication tools to identify acutely deteriorating patients. In the past few years, there has been a proliferation of studies that describe the development and validation of novel machine learning-based EWS. Systematic reviews of published studies which focus on evaluating performance of both well-established and novel EWS have shown conflicting conclusions. A possible reason is the heterogeneity in validation methods applied. In this review, we aim to examine the methodologies and metrics used in studies which perform EWS validation.

**Methods:**

A systematic review of all eligible studies from the MEDLINE database and other sources, was performed. Studies were eligible if they performed validation on at least one EWS and reported associations between EWS scores and inpatient mortality, intensive care unit (ICU) transfers, or cardiac arrest (CA) of adults. Two reviewers independently did a full-text review and performed data abstraction by using standardized data-worksheet based on the TRIPOD (Transparent reporting of a multivariable prediction model for individual prognosis or diagnosis) checklist. Meta-analysis was not performed due to heterogeneity.

**Results:**

The key differences in validation methodologies identified were (1) validation dataset used, (2) outcomes of interest, (3) case definition, time of EWS use and aggregation methods, and (4) handling of missing values. In terms of case definition, among the 48 eligible studies, 34 used the patient episode case definition while 12 used the observation set case definition, and 2 did the validation using both case definitions. Of those that used the patient episode case definition, 18 studies validated the EWS at a single point of time, mostly using the first recorded observation. The review also found more than 10 different performance metrics reported among the studies.

**Conclusions:**

Methodologies and performance metrics used in studies performing validation on EWS were heterogeneous hence making it difficult to interpret and compare EWS performance. Standardizing EWS validation methodology and reporting can potentially address this issue.

## Background

Early warning scores (EWS) are simple tools to help detect clinical deterioration to improve patient safety in hospitals. EWS are often implemented as part of a wider early warning system, also known as “rapid response system”, whereby detection of a likely deterioration will trigger an alert or pre-planned escalation of care by healthcare providers [1, 2]. These EWS use objective parameters such as vital signs and laboratory results, and may include subjective parameters (e.g. “nurses’ worry”) [3] as input; and then output an integer score. A higher score generally indicates a higher likelihood of clinical deterioration, but is not a direct estimate of risk.

The first EWS was published in 1997 [4], and the concept gradually gained traction with the National Institute for Health and Clinical Excellence (NICE) recommending the use of early warning systems to monitor all adult patients in acute hospital setting in a 2007 guideline [5]. Currently there are many different EWS that have become available and are routinely used in hospitals globally, including USA, UK, Netherlands, Denmark and South Korea [6–8]. The recent advancements in machine learning (ML) have also opened up a new paradigm of novel EWS development, using ML techniques such as random forests and deep neural networks, giving rise to arguably better EWS [9–11].

As EWS have an impact on patient care, it is critical that they are rigorously validated [12]. In this regard, several systematic reviews have already looked at the performance of various EWS [6–8]. Notably, these systematic reviews drew conflicting conclusions about EWS performance – Smith ME et al. concluded that EWS perform well, while Gao et al. and Smith GB et al. found conflicting and unacceptable performance. A possible reason for this disagreement is a lack of consistency in the methods used to validate EWS [8].

An example of difference in validation methods of EWS is between a study by researchers from Google and another study that validated the National Early Warning Score (NEWS) [13, 14]. Although both study teams validated their respective EWS ability to predict inpatient mortality, the former validated its EWS being used once, at the start of the admission (AUROC 0.93–0.94), while the latter validated their EWS using its score for every observation set of vitals measured for the entire admission (AUROC 0.89–0.90). In terms of case definition, we consider the former to have used the “patient episode” definition, while the latter used the “observation set” definition. Case definition is one of several differences in validation methods.

The aim of this review is to examine the different methodologies and performance metrics used in the validation of EWS so that readers will pay attention to specific aspects of the validation when making comparisons between EWS performance from published studies. As far as we are aware, there has not been any similar work done before. It is not this review’s intention to identify better performing EWS or to perform quality or bias assessment of the studies.

## Methods

### Search strategy

We used PubMed to search the MEDLINE database from inception to 22 Feb 2019 for studies of EWS in adult populations. We used the keywords “early warning score”, “predict”, “discriminate”, and excluded “paediatrics”, “children” and “systematic review”. For completeness, we also sought to include publications that we found but were not returned in the PubMed search. This involved looking at studies from other EWS review papers [6–8], and consulting with experts.

To assess the validation of EWS, we included only articles that performed validation on at least one EWS, in which investigators reported associations between EWS scores and inpatient mortality, intensive care unit (ICU) transfers, or cardiac arrest (CA). Systematic review papers were excluded as they lacked granularity in description of the data handling and statistical analysis. We also excluded studies which did prospective validation whereby the EWS was already in operation to influence care decisions and impact patient outcomes. In these cases, the validation did not purely evaluate the discriminative ability of the EWS, but also included other factors such as staff compliance and availability of rapid response resources.

Investigators then reviewed titles and abstracts of citations identified through literature searches, and eligible articles were selected for full-text review and data abstraction.

### Data abstraction

Pre-defined data for abstraction was largely based on the TRIPOD Checklist for Prediction Model Validation [15], with some added elements which the study team felt were pertinent to EWS.

A full-text review of each eligible study was performed by two investigators independently. Data for abstraction included the specific EWS validated, validation dataset used, number of subjects, population characteristics, outcome of interest (inpatient mortality, ICU transfer, cardiac arrest), method of validation (case definition, time of EWS use, type of aggregation for methods with multiple scores), method of handling missing values, and reported metrics. For discrepancies in the abstracted data, the investigators would perform a repeat review of the paper together to reach a consensus.

## Results

**Fig. 1:**
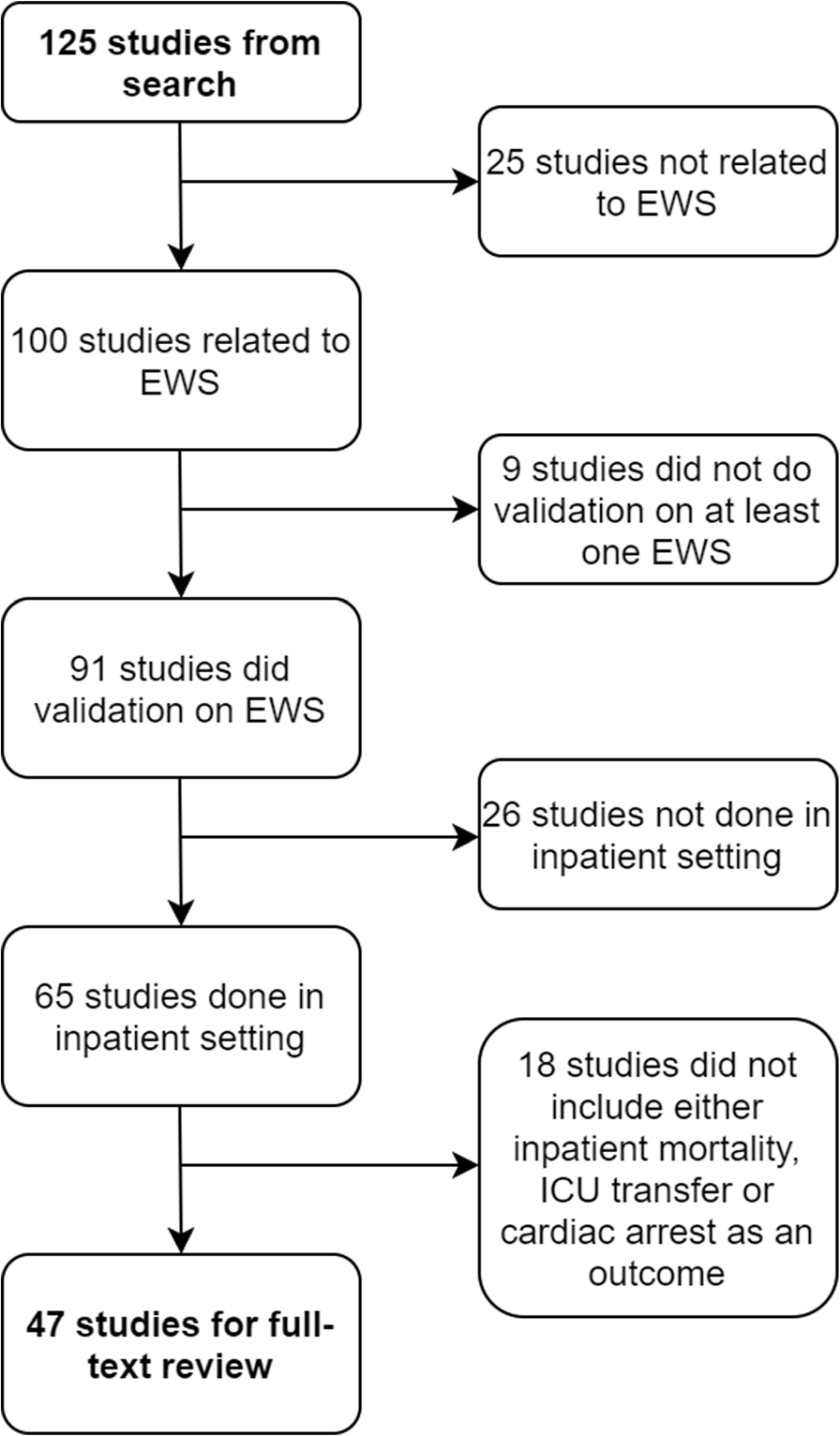
Flow chart describing inclusion of articles for full-text review from search result list

The PubMed search yielded a list of 125 study abstracts. From reviewing the study abstracts, 47 studies were selected for full-text review (Fig. 1). Of these, we excluded a further 12 studies – 11 (unable to access full study article) and 1 (full study article in Korean, only abstract in English). We included 13 additional relevant studies that we found from review papers and from consulting with experts. In total, 48 studies were included in the final review [3, 9, 11, 14, 16–59].

A summary table of the selected studies and data abstracted are found in Table 1 (see Additional file 1). 8 of the 48 studies were published in 2018 or later. Majority of the study populations were from UK (23) and USA (10), with 5 from South Korea and one each from Canada, China, Denmark, Hong Kong, Israel, Italy, Netherlands, Singapore, Sweden and Turkey.

Altogether, there were 54 unique EWS that were reviewed by the different studies, excluding the 33 other EWS assessed in the study by Smith in 2013 [14], and the 44 MET criteria assessed in the study by Smith in 2016 [27]. The most reviewed EWS were the Modified Early Warning Score (MEWS) and National Early Warning Score (NEWS), which were included in 22 and 16 studies respectively.

### Validation dataset

16 of the studies performed an internal validation, where a proportion of the entire study dataset was used to develop the EWS (training set), with the remaining proportion was used for validation (validation set) [9, 11, 17, 18, 20, 22, 24, 28, 32, 34, 35, 38, 39, 41, 44, 46, 57]. Varying proportion sizes were used for the validation set ranging from 25.0 to 100%. The other studies did an external validation with a study population different from that used to develop the EWS.

The study size used ranged from 43 to 269,999. Slightly over half (25 of 48) of the studies were performed on general admission cases, with the others focused on populations with specific conditions (e.g. chorioamnionitis [48], community-acquired pneumonia [31, 50, 51]), patients admitted to a certain specialty (e.g. Obstetrics [39], Haematology [28]), or only a subset of the general admission population (e.g. those reviewed by MET [33, 56], those with NEWS ≥1 [21]).

### Outcomes of interest

**Fig. 2:**
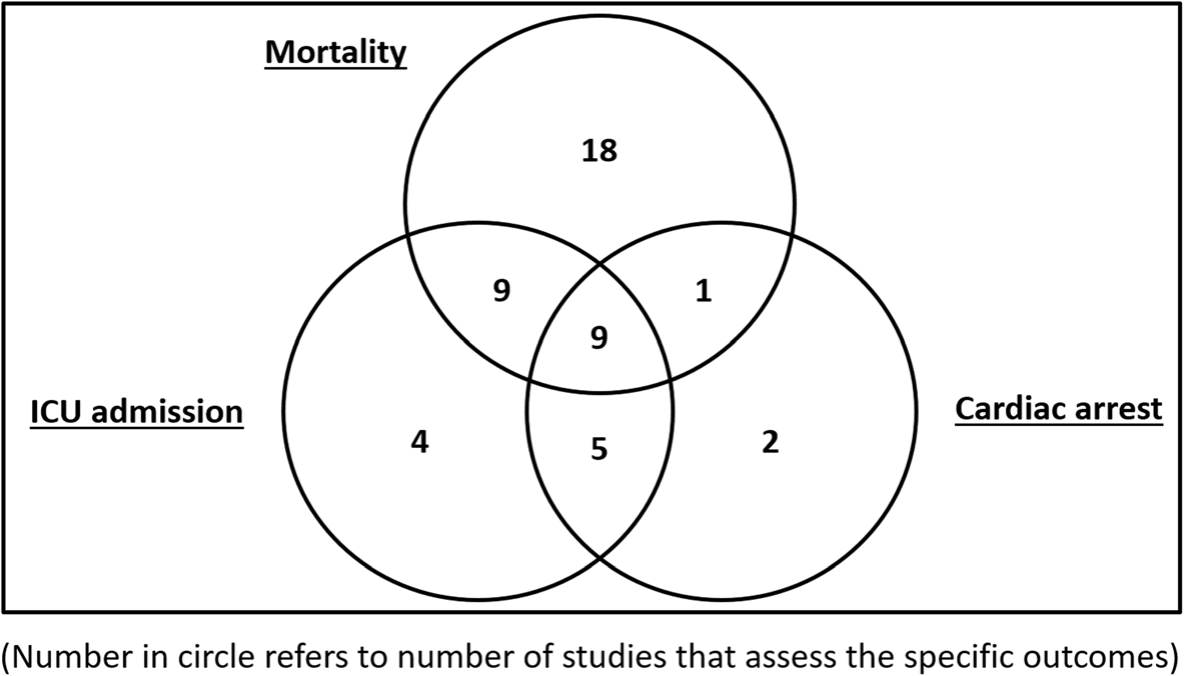
Summary of studies with various combinations of outcomes

All the studies included at least one of the outcomes of: inpatient mortality, ICU transfer or cardiac arrest, or a combination of them (Fig. 2).

For the 24 studies that evaluated more than one outcome, 17 studies validated EWS using a composite of all the outcomes as the endpoint, while the others validated EWS for each of the individual type of outcomes.

### Case definition, time of EWS use and aggregation method

**Fig. 3:**
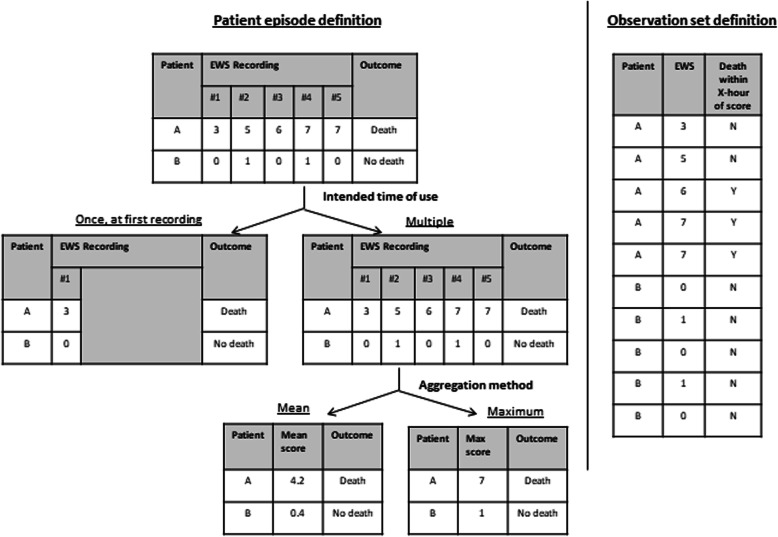
Illustration of how different case definition affect EWS validation for 2 patients

There were two different ways a case was defined – a patient episode or an observation set – and this definition had impact on the subsequent validation steps (Fig. 3). The patient episode definition considered an entire admission as a single case and used either a single or multiple recordings of vital signs and other parameters from the admission, while the observation set definition considered each observation set of vital signs and other parameter recordings from the same admission as independent of one another.

In the observation set definition, recordings from each observation set would be used to compute a score to evaluate the EWS association with an outcome within a certain time window from the time of recording of the observation set. In the patient episode definition, because multiple recordings were available, study teams either chose to use a single score, or multiple scores to validate the EWS. This reflected how study teams intended for the EWS to be used in practice, so we termed this component as “time of EWS use”. If the time of EWS use was multiple, then the series of scores would be aggregated in evaluating the EWS with the outcome of the patient episode.

Among the 48 studies, 34 used the patient episode method while 12 used the observation set method, and 2 did the validation using both methods.

Of the studies that used the patient episode method, 18 studies used a single point of time score to validate the EWS, most of which used the first recorded observation. For the 18 studies that used multiple recordings, 13 studies used use the maximum score as to aggregate the scores for each patient episode to compare with the outcomes. Most studies used all recordings from the patient episode, but there were 2 studies that excluded readings just prior to outcome to account for the “predictive” ability of the EWS [28, 44].

For the studies that used the observation set method, all of them used EWS values generated within the 0-24 hour time window prior to outcome to validate the EWS, with the exception of one study [11] that used EWS values within the 30 minute to 24 hour time window.

### Handling of missing values

As the validation datasets were obtained from real-world data, missing values were inevitable. In general, there were two ways the missing values were handled – either exclude or impute values. 19 studies excluded the missing values, 15 used imputations, 4 used a combination of both methods, and in 10 studies it was not stated. There were a variety of imputation methods used. The most commonly used were imputing a value from the last observation (6 studies), imputing a median value (3 studies), a combination of the last observation and median (5 studies) and imputing with a normal value (4 studies). There were also some sophisticated imputation methods, such as using random forest [17] and multiple imputation [23].

### Performance metrics

For performance metrics, we grouped them into two types – discrimination and calibration metrics. Discrimination is the measure of the EWS ability to differentiate significantly between cases with outcome and cases without outcome. Calibration is an evaluation of the extent to which estimated probabilities of the EWS scores agree with observed outcome rates [60]. Where an integer EWS was validated, it would not be possible to assess calibration.

The most commonly reported discrimination metric was the area under the curve of the receiver operating characteristics (AUROC). Only 6 studies did not use this metric. Twenty-two studies reported using any one of sensitivity, specificity, or predictive value (positive and negative). A lesser used alternative to the AUROC was the area under the precision-recall curve (AUPRC) which was used in two more recent studies by Kwon et al [11] and Watkinson et al [20]. The authors reasoned that this is a more suitable metric for verifying false-alarm rates with varying sensitivity.

The EWS efficiency curve was another measure used to visualize the discriminatory ability of EWS in 8 studies. The EWS efficiency curve was first introduced in the study by Smith et al. to provide a graphical depiction of the proportion of triggers that would be generated at varying EWS scores [13].

Six studies performed a statistical test of model calibration. Four used the Hosmer-Lemeshow goodness-of-fit test, one calculated the calibration slope and one used both metrics. Another six studies did not perform a statistical test of calibration, but provided a visualization of the model calibration.

## Discussion

Studies that validate EWS used a wide variety of validation methods and performance metrics [4, 9, 11, 14, 16–45]. Given that these variations have a bearing on EWS performance measurement, one should be mindful of them when interpreting and comparing bottom-line metrics, like AUROC values.

While the TRIPOD checklist for prediction model validation provides a standardized framework for multivariable predictive model validation reporting [15], it lacks the finer details for EWS which are more multi-faceted than typical prediction models. Unlike clinical prediction tools, EWS are unique in that they may be intended for use at multiple time-points over a patient episode. Some key differences in validation methodology we found in our review, and propose EWS evaluators take note of, are the validation dataset, outcomes of interest, case definition, time of EWS use, aggregation method, and handling of missing values. These differences could explain the reason for conflicting opinion on whether EWS perform well or otherwise [6–8].

In terms of EWS performance reporting, our review also had similar findings from previous reviews, that studies tended to give more prominence to discrimination and have rarely assessed model calibration [12, 61]. We concur with the TRIPOD recommendation that both discrimination and calibration should be considered when judging a model’s accuracy [15]. Also, this review found some reporting metrics that can be considered as promising alternatives to AUROC in EWS performance reporting. The AUPRC mentioned earlier is one of them. It was noted to be suitable for verifying false-alarm rates with varying sensitivity [21, 22]. Another would be to exclude measurements within a time window just prior to outcome, to account for the “predictive” ability of the EWS [21, 27, 44].

We acknowledge that a limitation of our review may be the fairly narrow search strategy to include EWS studies with keywords “predict” and “discriminate”, and thus might have unwittingly excluded other studies that performed EWS validation. Future reviews may consider broadening the scope of the initial search.

## Conclusions

Current EWS validation methods are heterogenous and this probably contributes to conflicting conclusions regarding their ability to discriminate or predict the patients at risk of clinical deterioration. A standardized method of EWS validation and reporting can potentially address this issue.

## Supplementary information

**Additional file 1: Table S1.**. Detailed summary of the 48 selected studies, with selected data for abstraction.

## Data Availability

All data used in the publication of this work were obtained from published studies. The abstracts for these studies are available in the MEDLINE database found on PubMed.

## Abbreviations

APACHE: Acute physiology and chronic health evaluation,
APPROVE: Accurate prediction of prolonged ventilation score
ASSIST: Assessment score for sick patient identification and step-up in treatment
AUPRC: Area under the precision-recall curve
AUROC: Area under the curve of the receiver operating characteristics
AWTTS: Aggregate weighted track and trigger systems
CA: Cardiac arrest
CARM: Computer aided risk of mortality
CART: Cardiac risk assessment triage
cCEWS: Continuously-recorded centile-based EWS
eCART: Electronic cardiac arrest risk triage
ED: Emergency Department
EEC: EWS Efficiency curve
EWS: Early warning score
LDTEWS: Lab decision-tree early warning score
LEWS: Leed’s early warning score
GI-EWS: Gastrointestinal EWS
GMEWS: Global Modified EWS
LOCF: Last observation carried forward
H: Hour
HL: Hosmer-Lemeshow test
ICU: Intensive care unit
LR: Likelihood ratio
MACHP: Mean alarm count per patient per hour
mCEWS: Manually-recorded centile-based EWS
MEDS: Mortality in Emergency Department Sepsis
MET: Medical Emergency Team
MEOWS: Modified early obstetric warning score
MEWS: Modified early warning score
ML: Machine learning
NEWS: National early warning score
NICE: National Institute for Health and Clinical Excellence
NPV: Negative predictive value
NRI: Net reclassification index
OR: Odds ratio
PARS: Patient-at-risk score
PIRO: Predisposition/infection/response/organ dysfunction
PMEWS: Pandemic medical early warning score
PPV: Positive predictive value
PSI: Pneumonia severity index
qSOFA: Quick Sequential related organ failure assessment
ROC: Receiver operating characteristics
RAPS: Rapid acute physiology score
REMS: Rapid emergency medicine score
RRT: Rapid response team
SAPS: Simplified acute physiology score
SCS: Simple clinical score
SCT: Stem cell transplant
SEWS: Standardized EWS
SIRS: Systemic inflammatory response syndrome
SOFA: Sequential organ failure assessment
TRIPOD: Transparent reporting of a multivariable prediction model for individual prognosis or diagnosis
ViEWS: VitalPac EWS
YI: Youden’s index

## References

[1] DeVita MA, Hillman K (2018). Why RRS? Where RRS?. Crit Care Clin.

[2] Alam N, Hobbelink EL, van Tienhoven AJ, van de Ven PM, Jansma EP, Nanayakkara PW (2014). The impact of the use of the Early Warning Score (EWS) on patient outcomes: a systematic review. Resuscitation.

[3] Douw G, Huisman-de Waal G, van Zanten AR, van der Hoeven JG, Schoonhoven L (2016). Nurses' 'worry' as predictor of deteriorating surgical ward patients: A prospective cohort study of the Dutch-Early-Nurse-Worry-Indicator-Score. Int J Nurs Stud.

[4] Morgan RJM, Williams F, Wright MM (1997). An early warning scoring system for detecting developing critical illness. Clin Intensive Care.

[5] National Institute for Health and Clinical Excellence. Acute ill patients in hospital: recognition of and response to acute illness in adults in hospital. In: NICE clinical guideline No. 50. London; 2007. 21204323

[6] Gao H, McDonnell A, Harrison DA (2007). Systematic review and evaluation of physiological track and trigger warning systems for identifying at-risk patients on the ward. Intensive Care Med.

[7] Smith GB, Prytherch DR, Schmidt PE (2008). Review and performance evaluation of aggregate weighted ‘track and trigger’ systems. Resuscitation.

[8] Smith ME, Chiovaro JC, O’Neil M (2014). Early warning system scores for clinical deterioration in hospitalized patients: a systematic review. Ann Am Thorac Soc.

[9] Churpek MM (2016). Multicenter Comparison of Machine Learning Methods and Conventional Regression for Predicting Clinical Deterioration on the Wards. Crit Care Med.

[10] Xu M, Tam B (2015). A protocol for developing early warning score models from vital signs data in hospitals using ensemble of decision tress. BMJ Open.

[11] Kwon JM, Lee Y, Lee Y, Lee S, Park J (2018). An Algorithm Based on Deep Learning for Predicting In-Hospital Cardiac Arrest. J Am Heart Assoc.

[12] Gerry S (2017). Early warning scores for detecting deterioration in adult hospital patients: a systematic review protocol. BMJ Open.

[13] Rajkomar A, Oren E, Chen K (2018). Scalable and accurate deep learning with electronic health records. NPJ Digit Med.

[14] Smith GB (2013). The ability of the National Early Warning Score (NEWS) to discriminate patients at risk of early cardiac arrest, unanticipated intensive care unit admission, and death. Resuscitation.

[15] Collins GS (2015). Transparent reporting of a multivariable prediction model for individual prognosis or diagnosis (TRIPOD): The TRIPOD statement.

[16] Lim WT (2019). Use of the National Early Warning Score (NEWS) to Identify Acutely Deteriorating Patients with Sepsis in Acute Medical Ward. Ann Acad Med Singap.

[17] Dziadzko MA (2018). Multicenter derivation and validation of an early warning score for acute respiratory failure or death in the hospital. Crit Care.

[18] Faisal M (2018). Development and validation of a novel computer-aided score to predict the risk of in-hospital mortality for acutely ill medical admissions in two acute hospitals using their first electronically recorded blood test results and vital signs: a cross-sectional study. BMJ Open.

[19] Hydes TJ (2018). National Early Warning Score Accurately Discriminates the Risk of Serious Adverse Events in Patients With Liver Disease. Clin Gastroenterol Hepatol.

[20] Redfen OC (2018). Predicting in-hospital mortality and unanticipated admissions to the intensive care unit using routinely collected blood tests and vital signs: Development and validation of a multivariable model. Resuscitation..

[21] Spångfors M, et al. The National Early Warning Score predicts mortality in hospital ward patients with deviating vital signs: A retrospective medical record review study. J Clin Nurs. 2018;5. 10.1111/jocn.14728 30516860

[22] Watkinson PJ (2018). Manual centile-based early warning scores derived from statistical distributions of observational vital-sign data. Resuscitation..

[23] Goulden R (2018). qSOFA, SIRS and NEWS for predicting inhospital mortality and ICU admission in emergency admissions treated as sepsis. Emerg Med J.

[24] Kim WY (2017). A risk scoring model based on vital signs and laboratory data predicting transfer to the intensive care unit of patients admitted to gastroenterology wards. J Crit Care.

[25] Tirotta D (2017). Evaluation of the threshold value for the modified early warning score (MEWS) in medical septic patients: a secondary analysis of an Italian multicentric prospective cohort (SNOOPII study). QJM..

[26] Delgado-Hurtado JJ (2016). Emergency department Modified Early Warning Score association with admission, admission disposition, mortality, and length of stay. J Community Hosp Intern Med Perspect.

[27] Durusu Tanrıöver M (2016). Daily surveillance with early warning scores help predict hospital mortality in medical wards. Turk J Med Sci.

[28] Hu SB (2016). Prediction of Clinical Deterioration in Hospitalized Adult Patients with Hematologic Malignancies Using a Neural Network Model. PLoS One.

[29] Kovacs C (2016). Comparison of the National Early Warning Score in non-elective medical and surgical patients. Br J Surg.

[30] Smith GB (2016). A Comparison of the Ability of the Physiologic Components of Medical Emergency Team Criteria and the U.K. National Early Warning Score to Discriminate Patients at Risk of a Range of Adverse Clinical Outcomes. Crit Care Med.

[31] Jo S (2016). Validation of modified early warning score using serum lactate level in community-acquired pneumonia patients. The National Early Warning Score-Lactate score. Am J Emerg Med.

[32] Liu FY (2015). A prospective validation of National Early Warning Score in emergency intensive care unit patients at Beijing. Hong Kong J Emerg Med.

[33] Yoo JW (2015). A combination of early warning score and lactate to predict intensive care unit transfer of inpatients with severe sepsis/septic shock. Korean J Intern Med.

[34] Churpek MM (2014). Multicenter development and validation of a risk stratification tool for ward patients. Am J Respir Crit Care Med.

[35] Churpek MM (2014). Using electronic health record data to develop and validate a prediction model for adverse outcomes in the wards. Crit Care Med.

[36] Kim WY (2015). Modified Early Warning Score Changes Prior to Cardiac Arrest in General Wards. PLoS One.

[37] Yu S (2014). Comparison of risk prediction scoring systems for ward patients: a retrospective nested case-control study. Crit Care.

[38] Badriyah T (2014). Decision-tree early warning score (DTEWS) validates the design of the National Early Warning Score (NEWS). Resuscitation..

[39] Carle C (2013). Design and internal validation of an obstetric early warning score: secondary analysis of the Intensive Care National Audit and Research Centre Case Mix Programme database. Anaesthesia..

[40] Corfield AR (2013). Utility of a single early warning score in patients with sepsis in the emergency department. Emerg Med J.

[41] Jarvis SW (2013). Development and validation of a decision tree early warning score based on routine laboratory test results for the discrimination of hospital mortality in emergency medical admissions. Resuscitation..

[42] Romero-Brufau S (2014). Widely used track and trigger scores: are they ready for automation in practice?. Resuscitation..

[43] Alrawi YA (2013). Predictors of early mortality among hospitalized nursing home residents. QJM..

[44] Churpek MM (2012). Derivation of a cardiac arrest prediction model using ward vital signs. Crit Care Med.

[45] Cooksley T (2012). Effectiveness of Modified Early Warning Score in predicting outcomes in oncology patients. QJM..

[46] Kellet J (2013). Changes and their prognostic implications in the abbreviated Vitalpac™ early warning score (ViEWS) after admission to hospital of 18,853 acutely ill medical patients. Resuscitation..

[47] Ghanem-Zoubi NO (2011). Assessment of disease-severity scoring systems for patients with sepsis in general internal medicine departments. Crit Care.

[48] Lappen JR (2010). Existing models fail to predict sepsis in an obstetric population with intrauterine infection. Am J Obstet Gynecol.

[49] Prytherch DR (2010). ViEWS – Towards a national early warning score for detecting adult inpatient deterioration. Resuscitation.

[50] Barlow G (2007). The CURB65 pneumonia severity score outperforms generic sepsis and early warning scores in predicting mortality in community-acquired pneumonia. Thorax.

[51] Challen K (2007). Physiological-social score (PMEWS) vs. CURB-65 to triage pandemic influenza: a comparative validation study using community-acquired pneumonia as a proxy. BMC Health Serv Res.

[52] von Lilienfeld-Toal M (2007). Observation-Based Early Warning Scores to Detect Impending Critical Illness Predict In-Hospital and Overall Survival in Patients Undergoing Allogeneic Stem Cell Transplant. Biol Blood Marrow Transpl.

[53] Kellet J (2006). The Simple Clinical Score predicts mortality for 30days after admission to an acute medical unit. Q J Med.

[54] Lam TS (2006). Validation of a Modified Early Warning Score (MEWS) in emergency department observation ward patients. Hong Kong J Emerg Med.

[55] Subbe CP (2006). Validation of physiological scoring systems in the accident and emergency department. Emerg Med J.

[56] Goldhill DR (2005). A physiologically-based early warning score for ward patients: the association between score and outcome. Anaesthesia.

[57] Olsson T (2004). Rapid Emergency Medicine score: a new prognostic tool for in-hospital mortality in nonsurgical emergency department patients. J Intern Med.

[58] Hodgetts TJ (2002). The identification of risk factors for cardiac arrest and formulation of activation criteria to alert a medical emergency team. Resuscitation.

[59] Subbe CP (2001). Validation of a modified Early Warning Score in medical admissions. Q J Med.

[60] Steyerberg E (2010). Assessing the performance of prediction models: a framework for some traditional and novel measures. Epidemiology..

[61] Van Calster B (2019). Calibration: the Achilles heel of predictive analytics. BMC Med.

