# Correction: Early warning score validation methodologies and performance metrics: a systematic review

**DOI:** 10.1186/s12911-024-02795-7

**Published:** 2025-01-06

**Authors:** Andrew Hao Sen Fang, Wan Tin Lim, Tharmmambal Balakrishnan

**Affiliations:** 1 Bedok Polyclinic, SingHealth Polyclinics, Singapore, Singapore; 2 Department of Internal Medicine, Singapore General Hospital, Singapore, Singapore; 3 Department of Internal Medicine, Singapore General Hospital, Singapore, Singapore

**Correction to: Fang et al. BMC Medical Informatics and Decision Making (2020) 20:111**

10.1186/s12911-020-01144-8

Following the publication of the original article, a reader identified an incorrect citation in the “Performance metrics” section. The final sentence cited reference no. 13, whereas it should have cited reference no. 14 (*Smith GB, et al. The ability of the National Early Warning Score (NEWS) to discriminate patients at risk of early cardiac arrest, unanticipated intensive care unit admission, and death. Resuscitation. 2013;84(4):465–70*).

The author has confirmed this correction. Therefore, the correct sentence is as follows:

“The EWS efficiency curve was first introduced in the study by Smith et al. to provide a graphical depiction of the proportion of triggers that would be generated at varying EWS scores [**14**].”

We thank the reader for bringing this to our notice.

Meanwhile, the previously listed email address of the Corresponding Author has been inactive, thus it has been revised to andrew.fang@doctoranywhere.com.

The Original Article has been corrected.

