# Supplementary material for: Early warning score validation methodologies and performance metrics: a systematic review
Source: BMC Med Inform Decis Mak. 2020 Jun 18;20:111. doi: 10.1186/s12911-020-01144-8 (PMC7301346; doi:10.1186/s12911-020-01144-8)
Supplement: Supplementary file 1 — Additional file 1: Table S1.. Detailed summary of the 48 selected studies, with selected data for abstraction. [file 12911_2020_1144_MOESM1_ESM.docx]

Table 1. Detailed summary of the 48 selected studies, with selected data for abstraction

|  | **Study** | | | **EWS** | **Validation set** | | | **Outcome** | | | **Case definition** | **Time of EWS use** | | **Aggregation method** | **Missing values** | | **Metric** | | | |
| --- | --- | --- | --- | --- | --- | --- | --- | --- | --- | --- | --- | --- | --- | --- | --- | --- | --- | --- | --- | --- |
|  | **Author (First)** | **Year** | **Country** |  | **Relation to dataset for EWS development** | **Study Size (% used for validation)** | **Population Type** | **Mortality** | **ICU** | **CA** |  | **Once** | **Multiple** |  | **Exclude** | **Impute** | **Classification** | | | **Calibration** |
|  |  |  |  |  |  |  |  |  |  |  |  |  |  |  |  |  | **ROC** | **Sen/Sp/PPV/NPV** | **Others** |  |
| 1 | Lim | 2019 | Singapore | NEWS | External | 11,300 (100) | General | Y# (Within 24H) | Y# (Within 24H) | Y# (Within 24H) | Observation set |  |  |  | (Not stated) | (Not stated) | Y |  |  |  |
| 2 | Dziadzko | 2018 | USA | - APPROVE - MEWS   NEWS | Internal (for APPROVE); External (for NEWS and MEWS) | 68,775 (50.0) | General | Y |  |  | Patient episode |  | All recordings | First score to exceed pre-defined cut-off |  | Random forest | Y | Y |  |  |
| 3 | Faisal | 2018 | UK | CARM score | Internal | 73,851  (49.7) | General | Y |  |  | Patient episode | First recorded |  |  |  | Median | Y | Y |  | Calibration slope |
| 4 | Hydes | 2018 | UK | NEWS | External | 35,585 (100) | Liver disease | Y#  (Within 24H) | Y#  (Within 24H) | Y#  (Within 24H) | Observation set |  |  |  | Y |  | Y |  | EEC |  |
| 5 | Kwon | 2018 | South Korea | - Deep learning based EWS - Random forest EWS - Logistic regression EWS   MEWS | Internal, except for MEWS (External) | 52,131 (10.4) | General | Y  (Within 0.5-24H) |  | Y  (Within 0.5-24H) | Observation set |  |  |  |  | LOCF, Median | Y | Y | AUPRC, NRI, MACHP |  |
| 6 | Redfern | 2018 | UK | - LDTEWS:NEWS risk index - NEWS | Internal (for LDTEWS:NEWS); External (for NEWS) | 97,933 (38.1) | General | Y  (Within 24H) | Y (Within 24H) |  | Observation set |  |  |  |  | LOCF | Y |  |  | (Visually assessed) |
| 7 | Spångfors | 2018 | Denmark | - NEWS | External | 1,107 (100) | NEWS ≥1 | Y |  |  | Patient episode | First recorded |  |  | Y | LOCF | Y |  | OR |  |
| 8 | Watkinson | 2018 | UK | - mCEWS - cCEWS - NEWS | Internal, except for NEWS (External) | 65,548 (81.5) | General | Y#  (Within 24H) | Y#  (Within 24H) | Y#  (Within 24H) | Observation set |  |  |  | Y | Mean | Y |  | AUPRC |  |
| 9 | Goulden | 2017 | UK | - qSOFA - SIRS criteria - NEWS | External | 1,818 (100) | Suspected sepsis | Y | Y |  | Patient episode | First recorded |  |  |  | Regression, multiple imputation | Y | Y | LR |  |
| 10 | Kim | 2017 | South Korea | - EWS-GI - MEWS | Internal (for EWS-GI); External (for MEWS) | 1,219 (100) | GI disorders with MET activation |  | Y |  | Patient episode | At point of MET activation |  |  | (Not stated) | (Not stated) | Y | Y |  | HL χ2 |
| 11 | Tirotta | 2017 | Italy | MEWS | External | 526 (100) | Suspected sepsis | Y |  |  | Patient episode | First recorded |  |  | Y |  | Y | Y | LR |  |
| 12 | Churpek | 2016 | USA | Machine-learning based EWS (9 different types)MEWS | Internal, except for MEWS (External) | 269,999 (40.0) | General | Y#  (Within 24H) | Y#  (Within 24H) | Y#  (Within 24H) | Observation set |  |  |  |  | LOCF, median | Y |  | EEC | HL χ2, Calibration slope, Calibration intercept |
| 13 | Delgado-Hurtado | 2016 | USA | MEWS | External | 2,147 (100) | General (ED) | Y |  |  | Patient episode |  | All recordings | Mean, Maximum | Y |  |  |  | OR |  |
| 14 | Durusu | 2016 | Turkey | MEWS | External | 182 (100) | General | Y |  |  | Patient episode |  | All recordings | Maximum, Total, First, Last | Y |  | Y | Y |  |  |
| 15 | Hu | 2016 | USA | - Neural network EWS - ViEWS | Internal (for Neural network EWS); External (for ViEWS) | 565 (25.0) | Hematology unit admission |  | Y# | Y# | Patient episode |  | All recordings, up to 4H prior to outcome | (Not stated) |  | LOCF | Y | Y |  |  |
| 16 | Kovacs* | 2016 | UK | NEWS | External | 87,399 (100) | Surgical (non-elective and elective), Medical (non-elective and elective) | Y#  (Within 24H) | Y#  (Within 24H) | Y#  (Within 24H) | Observation set |  |  |  | Y |  | Y |  | EEC | (Distribution of cases by score) |
|  |  |  |  |  |  |  |  | Y# | Y# | Y# | Patient episode |  | All recordings | Random | Y |  | Y |  |  |  |
| 17 | Smith | 2016 | UK | - NEWS   MET criteria (44 different ones) | External | 103,998 (100) | General | Y#  (Within 24H) | Y#  (Within 24H) | Y#  (Within 24H) | Observation set |  |  |  | Y |  | Y | Y | EEC |  |
| 18 | Douw | 2015 | Nether-lands | Dutch-Early-Nurse-Worry-Indicator Score | External | 3,522 (100) | Surgical | Y# | Y# |  | Patient episode | First recording within 24 hours of outcome |  |  |  | LOCF | Y |  |  |  |
| 19 | Jo | 2015 | South Korea | - NEWS-lactate - NEWS - PSI - CURB-65 score | External | 553 (100) | Pneumonia | Y |  |  | Patient episode | First recorded |  |  | Y |  | Y | Y |  | HL χ2,  (Distribution of cases by score) |
| 20 | Liu | 2015 | China | - NEWS - MEWS | External | 551 (100) | Severe-acuity emergencies | Y  (Within 24H) |  |  | Patient episode | First recorded |  |  | Y |  | Y |  | OR, YI |  |
| 21 | Yoo | 2015 | South Korea | - MEWS-lactate - MEWS | External | 100 (100) | Sepsis (severe) seen by MET team |  | Y |  | Patient episode | At point of MET activation |  |  | Y |  | Y | Y |  |  |
| 22 | Churpek | 2014 | USA | - eCART score - MEWS | Internal (for eCART score); External (for MEWS) | 269,999 (40.0) | General | Y#  (Within 24H) | Y#  (Within 24H) | Y#  (Within 24H) | Observation set |  |  |  |  | LOCF, median | Y | Y |  |  |
| 23 | Churpek* | 2014 | USA | - Person-time multinomial logistic regression model - ViEWS | Internal (for Person-time multinomial logistic regression model); External (for ViEWS) | 59,301 (100) | General |  | Y  (Within 24H) | Y  (Within 24H) | Observation set |  |  |  |  | LOCF, median | Y |  |  |  |
|  |  |  |  |  |  |  |  |  | Y |  | Patient episode |  | All recordings | Maximum |  |  | Y | Y |  |  |
| 24 | Kim | 2014 | South Korea | - MEWS | External | 380 (100) | In-hospital CA, and then ROSC by MET |  |  | Y | Patient episode |  | 0-8H, 8-16H, and 16-24H before CA | Maximum (of each block) | Y |  |  |  | OR |  |
| 25 | Yu | 2014 | USA | - SOFA - PIRO score - ViEWS - SCS - MEDS - MEWS - SAPS-II - APACHE-II - REMS | External | 656 (100) | Infection | Y# | Y# |  | Patient episode |  | 0-12H, 12-24H, 24-48H, and 48-72H before outcome | Maximum (of each block) |  | LOCF, median | Y | Y | OR |  |
| 26 | Badriyah | 2013 | UK | - Decision-tree based EWS - NEWS | Internal (for Decision-tree based EWS); External (for NEWS) | 35,585 (100) | General | Y#  (Within 24H) | Y#  (Within 24H) | Y#  (Within 24H) | Observation set |  |  |  | (Not stated) | (Not stated) | Y |  | EEC | (Distribution of cases by score) |
| 27 | Carle | 2013 | UK | - Statistical EWS - Clinical EWS - MEOWS - Confidential Enquiry into Maternal Deaths EWS - NEWS | Internal (for Statistical EWS); External (for the rest) | 4,440 (49.5) | Obstetrics | Y |  |  | Patient episode |  | All recordings within 24H of outcome | Most abnormal of each parameter used to compute score |  | Median | Y |  |  |  |
| 28 | Corfield | 2013 | UK | - NEWS | External | 2003 (100) | Sepsis | Y# | Y# |  | Patient episode | First recorded |  |  | Y |  | Y | Y | OR |  |
| 29 | Jarvis | 2013 | UK | - LDTEWS | Internal | 86,472 (100) | General | Y |  |  | Patient episode | First recorded |  |  | Y |  | Y |  | EEC | (Distribution of cases by score) |
| 30 | Romeo-Brufau | 2013 | USA | - MEWS - SEWS - GMEWS - Worthing - ViEWS - NEWS - RRT activation criteria | External | 34,898 (100) | General |  | Y#  (Within 3, 8, 12, 24, and 36H) | Y#  (Within 3, 8, 12, 24, and 36H) | Observation set |  |  |  |  | LOCF | Y | Y |  |  |
| 31 | Smith | 2013 | UK | - NEWS - 33 other EWS | External | 35,585 (100) | General | Y#  (Within 24H) | Y#  (Within 24H) | Y#  (Within 24H) | Observation set |  |  |  | (Not stated) | (Not stated) | Y |  | EEC | (Distribution of cases by score) |
| 32 | Alrawi | 2012 | UK | MEWS | External | 314 (100) | Nursing home | Y  (Within 1^st^ week of adm) |  |  | Patient episode | First recorded |  |  | (Not stated) | (Not stated) |  |  | OR |  |
| 33 | Churpek | 2012 | USA | - CART score   MEWS | Internal (for CART score); External (for MEWS) | 47,427 (100) | General |  | Y | Y | Patient episode |  | All recordings, up to 30min prior to outcome | Maximum |  | LOCF | Y |  |  |  |
| 34 | Cooksley | 2012 | UK | MEWS | External | 840 (100) | Oncology, assessed by outreach team |  | Y |  | Patient episode | At point of Acute Oncology Nurse Specialist review |  |  | Y |  | Y |  |  |  |
| 35 | Kellett | 2012 | Canada | ViEWS | External | 18,853 (100) | General | Y |  |  | Patient episode |  | First 3 recordings | Differences between first and second, and first and third | Y |  |  |  | OR |  |
| 36 | Ghanem-Zoubi | 2011 | Israel | - MEWS - SCS - MEDS - REMS | External | 1,072 (100) | Sepsis | Y |  |  | Patient episode |  | All recordings, up to admission | Maximum | (Not stated) | (Not stated) | Y |  |  | HL χ2 |
| 37 | Lappen | 2010 | USA | - SIRS - MEWS | External | 913 (100) | Chorio-amnionitis | Y# | Y# |  | Patient episode |  | All recordings | Maximum |  | Impute with normal value |  | Y |  | (Distribution of cases by score) |
| 38 | Prytherch | 2010 | UK | - ViEWS - AWTTS (33 different ones) | External | 35,585 (100) | General | Y  (Within 0-24H, 6-24H, 8-24H and 18-24H) |  |  | Observation set |  |  |  | (Not stated) | (Not stated) | Y |  |  |  |
| 39 | Barlow | 2007 | UK | - CURB65 - CRB65 - SIRS - SEWS | External | 419 (100) | Community-acquired pneumonia | Y |  |  | Patient episode | First recorded |  |  | (Not stated) | (Not stated) | Y | Y |  |  |
| 40 | Challen | 2007 | UK | - PMEWS - CURB65 | External | 186 for mortality, 144 for ICU (100) | Community-acquired pneumonia | Y | Y |  | Patient episode | First recorded |  |  | Y (if 3 or more data missing) | Imputed with normal value (if one or two data missing) | Y |  |  |  |
| 41 | von Lilienfeld-Toal | 2007 | UK | - MEWS - PARS - LEWS | External | 43 (100%) | Adult recipients of SCT | Y |  |  | Patient episode |  | All recordings | Maximum |  | Imputed with normal value | Y | Y |  |  |
| 42 | Kellett | 2006 | UK | SCS | Internal | 9964 (32.4) | General | Y |  |  | Patient episode | First recorded |  |  | (Not stated) | (Not stated) | Y |  |  |  |
| 43 | Lam | 2006 | Hong Kong | MEWS | External | 425 (100) | General | Y# | Y# |  | Patient episode |  | All recordings | Maximum | Y |  | Y | Y | OR |  |
| 44 | Subbe | 2006 | UK | - MEWS - ASSIST - MET criteria | External | 151 (100) | General |  | Y |  | Patient episode | First recorded |  |  | Y |  |  | Y |  |  |
| 45 | Goldhill | 2005 | UK | PARS | External | 1047 (100) | Patients assessed by an intensive care outreach service | Y | Y |  | Patient episode |  | All recordings | (Not stated) | Y |  | Y |  |  |  |
| 46 | Olsson | 2004 | Sweden | - REMS - RAPS | Internal (for REMS); External for RAPS | 12,006 (50.0 for REMS, 100 for RAPS) | Non-surgical | Y |  |  | Patient episode | First recorded |  |  | Y | Imputed with normal value | Y |  | LR, OR | HL χ2 |
| 47 | Hodgetts | 2002 | UK | - MET criteria | Internal | 250 (100) | General |  |  | Y | Patient episode |  | All recordings | Cumulative | (Not stated) | (Not stated) | Y | Y | OR |  |
| 48 | Subbe | 2001 | UK | MEWS | External | 709 (100) | General |  | Y# | Y# | Patient episode |  | All recordings | Maximum | Y |  | Y |  | OR |  |

*Definition of abbreviations*: APACHE = Acute Physiology and Chronic Health Evaluation, APPROVE = Accurate Prediction of Prolonged Ventilation Score, ASSIST = Assessment Score for Sick patient Identification and Step-up in Treatment, AUPRC = Area under precision-recall curve, AWTTS = Aggregate weighted track and trigger systems, CARM = Computer aided risk of mortality, CART = Cardiac Risk Assessment Triage, cCEWS = continuously-recorded centile-based EWS, eCART = Electronic Cardiac Arrest Risk Triage, ED = Emergency Department, EEC = EWS Efficiency curve, GI-EWS = Gastrointestinal EWS, GMEWS = Global Modified EWS, H = hour, HL = Hosmer-Lemeshow test, LDTEWS = Lab decision-tree early warning score, LEWS = Leed’s early warning score, LOCF = Last observation carried forward, LR = Likelihood ratio, MACHP = Mean alarm count per patient per hour, mCEWS = manually-recorded centile-based EWS, MEDS = Mortality in Emergency Department Sepsis, MET = Medical Emergency Team, MEOWS = Modified Early Obstetric Warning Score, MEWS = Modified Early Warning Score, NPV = Negative predictive value, NRI = Net reclassification index, OR = Odds ratio, PARS = Patient-at-risk score, PIRO = Predisposition/Infection/Response/Organ Dysfunction, PMEWS = Pandemic Medical Early Warning Score, PPV = Positive predictive value, PSI = Pneumonia Severity Index, qSOFA = quick Sequential Related Organ Failure Assessment, RAPS = Rapid Acute Physiology Score, REMS = Rapid Emergency Medicine Score, RRT = Rapid Response Team, ROC = Receiver operating characteristic, SAPS = Simplified Acute Physiology Score, SCS = Simple Clinical Score, SCT = stem cell transplant, SEWS = Standardized EWS, SIRS = Systemic Inflammatory Response Syndrome, SOFA = Sequential Organ Failure Assessment, ViEWS = VitalPac EWS, YI = Youden’s index

*Performed validation using two different methods – patient episode and observation set.

#Performed validation on a combination of outcomes.
